# Pregnancy and reproductive healthcare among adolescent girls in the child welfare system

**DOI:** 10.3389/fped.2025.1708452

**Published:** 2025-12-10

**Authors:** Sarah Font, Beatriz De Araujo Silva, Dylan Jones

**Affiliations:** 1 Brown School of Social Work, Washington University in St. Louis, St. Louis, MO, United States; 2 School of Public Policy, Pennsylvania State University, University Park, PA, United States; 3 Department of Human Development and Family Studies, Pennsylvania State University, University Park, PA, United States

**Keywords:** child welfare, pregnancy, reproductive health, foster care, adolescence

## Abstract

**Objective:**

This study examines the prevalence and predictors of first pregnancy and the use of long-acting reversible contraceptives (LARCs) among adolescent girls aged 12–17 involved in the child welfare system.

**Methods:**

The study leverages linked longitudinal administrative data from the child welfare, juvenile legal, and Medicaid claims systems of Pennsylvania. The sample comprised 28,016 girls born between 2000 and 2005 who received Medicaid and were involved in the child welfare system during adolescence. Survival analysis was used to predict onset of first pregnancy and uptake of LARC.

**Results:**

By age 18, 17% percent of adolescents had experienced pregnant, with 18% having a healthcare encounter to receive LARCs. Foster care placement was not associated with pregnancy but was associated with increased use of LARCs. Substance use disorder, juvenile legal involvement, and recent suspected sexual abuse victimization were positively associated with pregnancy. There was a reciprocal association between LARCs and pregnancy; although any previous encounter for LARCs was negatively associated with pregnancy, uptake of LARCs also increased following a pregnancy.

**Conclusions:**

Despite broad reductions in teen pregnancy over time, adolescent girls with exposure to sexual abuse or engagement in substance use or delinquency face a persistently high risk for very early pregnancy. Medical providers and child welfare agencies need to coordinate service provision to address the sexual and reproductive health needs of at-risk teens.

## Introduction

Despite large and sustained declines in adolescent pregnancy and parenthood in the US (1), girls from socially disadvantaged backgrounds continue to have high rates of early pregnancy and motherhood, as well as other sexual and reproductive health concerns, such as sexually transmitted infections (STIs) (2). The involvement of the child welfare system (CWS) is a well-established risk factor for early pregnancy and motherhood (3–6), consistent with the harmful effects of exposure to maltreatment on high-risk sexual behavior and an intention and desire for early pregnancy (7). Adolescent mothers, in turn, are substantially more likely to become involved with CWS as perpetrators of maltreatment and to lose custody of their children (8). Of particular concern are pregnancies among minors—that is, adolescent girls who become pregnant or enter parenthood prior to reaching the age of majority. These pregnancies—the focus of this study—are especially likely to disrupt secondary education and may carry heightened risks of sexual assault or exploitation.

Much remains unknown about how to delay pregnancy among maltreated girls. Sexual abuse is a well-established risk factor for early pregnancy (9), yet children's exposure to sexual abuse may be under-documented in case files due to non-disclosure, underreporting, or lack of evidence (10, 11). Moreover, inadequate caregiver monitoring—a feature of child neglect—is a consistent predictor of high-risk sexual behavior (12). Several studies have assessed foster care placement as a possible risk factor, with conflicting conclusions (3, 4). Though underexplored in the literature, adolescent girls involved with both CWS and the juvenile legal system—a relatively large group (13)—may experience particularly high rates of early pregnancy, due to shared risk factors related to substance use and low parental monitoring (14, 15). In addition, there is limited knowledge about whether particular mental and behavioral health characteristics help to identify adolescent girls at highest risk for early pregnancy.

Preventive healthcare, including access to the most effective contraception options (e.g., long-acting reversible contraceptives, LARCs), is an important part of the care continuum for adolescents at risk of early pregnancy. LARCs include hormone-free intrauterine devices as well as hormonal intrauterine devices and implants inserted into the arm. They are reversible (via removal) but can protect against pregnancy for 3–12 years unless removed. Studies have demonstrated that education about and low-burden access to LARCs can reduce the incidence of teen pregnancy and parenthood (16, 17). However, a 2019 national survey found that 38.7% of females aged 15–19 used any contraceptive method but only 5.8% used LARCs (18). This is particularly concerning given the substantial share of unintended pregnancies that occur while using other contraceptive methods. Relative to other prescription contraception methods, LARCs can impose additional barriers due to the need for multiple appointments, limited availability of providers for insertion of the device, and confusion or lack of clarity regarding consent for minors (particularly those in CWS custody). Adolescent girls involved with CWS may face additional barriers to reliable use of daily or incident-based contraceptive methods. For example, residential instability can inhibit stable access to a provider and pharmacy. CWS youths also experience elevated rates of mental health and substance use problems, which may interfere with decision-making and medication compliance.

Overall, there is limited evidence on the prevalence and predictors of sexual health risks for CWS-involved adolescent girls and whether and how at-risk adolescent girls are receiving reproductive healthcare. This study follows a Pennsylvania cohort of adolescent girls (12–17 years) who were enrolled in Medicaid and had confirmed CWS cases (*N* = 28,016). Upon leveraging CWS, Medicaid claims, and juvenile legal records, the study will address the following three questions: (1) What share of CWS-involved adolescent girls experience pregnancy, parenthood, and other sexual health risks prior to age 18? (2) What share of CWS-involved adolescent girls access contraceptive services, particularly LARCs? (3) How do the risks of pregnancy and use of LARC vary by foster care experience, juvenile legal involvement, and mental and behavioral health characteristics, including psychiatric disorders and delinquency?

## Method

### Study site

In Pennsylvania, CWS responds to both child maltreatment and severe child behavioral health challenges, including truancy, substance use, and other conditions that endanger the welfare of the child and are not manageable by the caregivers of the child. Child abuse, which is narrowly defined in Pennsylvania relative to other states (i.e., does not include general neglect), is handled by Child Protective Services and all other family concerns are handled by the state's alternative response, General Protective Services. Both types of cases can result in court involvement and placement. Pennsylvania expanded Medicaid coverage under the Affordable Care Act, with implementation effective since January 2015. As in all states, children in foster care are categorically eligible for Medicaid. Adolescents are legally permitted to access contraception and pregnancy-related care without parental involvement or consent (19), but physicians may be reluctant to offer LARCs to minors (20).

### Data and sample

The study sample was constructed from CWS records, Medicaid claims, and juvenile legal records from the state of Pennsylvania. The data include all adolescent girls (12–17 years of age) with a confirmed CWS case between 2015 and 2021 (for abuse, neglect, or child behavior concerns) in Pennsylvania. We restricted the sample to girls born between 2000 and 2005 who were enrolled in Medicaid for at least 2 years during the observation period. Our Medicaid linkage (and thus the period of outcome measurement) extended from 2012 to 2019. The final sample comprised 28,016 individuals.

### Measures

#### Sexual and reproductive health measures

*Pregnancy* was identified based on ICD 9 and ICD 10 codes in the Medicaid claims data (see Appendix A for code lists). The start of pregnancy was estimated based on the initial claim date associated with the pregnancy. *LARC* was defined using a combination of diagnostic and procedure codes, as described in Appendix A. *Other contraceptive consultations* were identified based on ICD codes for contraceptive management, excluding claims related to LARCs. We caution that, because pharmacy claims data were unavailable, we cannot ascertain whether these consultations resulted in prescribed contraceptives, such as oral birth control pills. For descriptive purposes, we report on the following additional measures, also identified from Medicaid claims: STI, diagnostic indicators of high-risk sexual behavior, and live birth.

#### Child welfare and juvenile legal records

Three time-varying CWS allegation variables were derived: (1) a confirmed report within the past 6 months related to child behavioral concerns (e.g., truancy, substance use, and general behavioral health problems); (2) a confirmed report within the past 6 months related to sexual abuse; and (3) a confirmed report within the past 6 months for any other child welfare concern. Foster care status was a three-category time-varying measure: never experienced foster care to date; currently in foster care; and previously discharged from foster care (to reunification, adoption, guardianship, or another setting). Juvenile legal involvement was measured dichotomously, equal to 1 if the adolescent had a delinquency referral in the current month and 0 otherwise.

#### Other covariates

We included a set of demographic and health control variables to account for potential confounders. Time-stable demographic measures were race/ethnicity (White, Black, Hispanic, and other) and birth cohort (year of birth variable, ranging from 2000 to 2005). Mental health indicators were dichotomous, non-mutually exclusive, ever-to-date measures for the following diagnoses: substance use disorders, post-traumatic stress disorder (PTSD), disruptive behavior disorders, attention-deficit/hyperactivity disorder (ADHD), mood disorders, anxiety disorders, autism spectrum, intellectual disabilities, and other psychiatric or developmental disorders.

### Analytic approach

We employed Cox proportional hazards models to analyze two main outcomes: adolescent first pregnancy and first LARC consultation among adolescent girls. The data were structured by person-month. Adolescent girls became at risk of pregnancy and use of first LARC at age 12. However, they entered observation at whichever was later: their 12th birthday or January 2015 (the earliest date of complete CWS report data). The cohort was followed until whichever came first: 18th birthday or December 2019 (end of our Medicaid records). A lagged version of LARC was included in the pregnancy model, and a lagged measure of pregnancy was included in the LARC model. Both models are produced for the full sample and then within subsamples disaggregated by race/ethnicity. All analyses were conducted using Stata 18.

## Results

### Sample description

Table 1 provides a description of the sample. The majority of the individuals were non-Hispanic White (59.6%), followed by non-Hispanic Black (24.3%) and Hispanic (12.8%). Thirteen percent had experienced foster care during adolescence, with an additional 5% having been in foster care earlier in childhood and not during adolescence. One-sixth of the sample (16.8%) experienced involvement in the juvenile legal system. Seventeen percent had a CWS allegation related to sexual abuse victimization during the observation period, whereas 44.2% had CWS allegations related to their own behavioral health.

**Table 1:** Sample description.

	Full sample		Ever pregnant		Ever LARC	
	*N*	Col. %	*N*	Row %	*N*	Row %
Total *N* (%)	28,016		2,621	9.36	2,610	9.32
Race/ethnicity						
White	16,710	59.64	1,275	7.63	1,574	9.42
Black	6,823	24.35	885	12.97	644	9.44
Hispanic	3,572	12.75	402	11.25	343	9.60
Other race/ethnicity	911	3.25	59	6.48	49	5.38
Types of CWS allegations						
Sexual abuse	4,775	17.04	473	9.91	576	12.06
Child behavioral issues	12,395	44.24	1,555	12.55	1,423	11.48
Foster care	6,734	24.04	965	14.33	933	13.86
Before age 12	1,296	4.63	171	13.19	167	12.89
During ages 12–17	3,859	13.77	649	16.82	635	16.46
Juvenile legal involvement	4,712	16.82	905	19.21	776	16.47
Mental health disorder diagnoses						
Substance use	5,156	18.40	1,097	21.28	1,024	19.86
PTSD	6,152	21.96	783	12.73	966	15.70
Conduct/oppositional defiant	7,988	28.51	1,109	13.88	1,107	13.86
ADHD	7,944	28.36	899	11.32	1,049	13.20
Mood	14,584	52.06	1,737	11.91	1,934	13.26
Anxiety	10,618	37.90	1,136	10.70	1,416	13.34

Turning to mental health conditions, a majority of adolescent girls had a mood disorder diagnosis (52.1%), with substantial shares also diagnosed with anxiety (37.9%), ADHD (28.4%), and disruptive disorders (e.g., conduct or oppositional defiant disorder; 28.5%). More than one in five adolescent girls had a PTSD diagnosis, and 18.4% had a substance use disorder diagnosis.

Rates of pregnancy and use of LARCs were elevated among adolescent girls with current or prior foster care placement, juvenile legal involvement, and mental health diagnoses. Rates of pregnancy were also elevated among Black and Hispanic adolescent girls.

### Prevalence of sexual and reproductive health indicators

**Figure 1:**
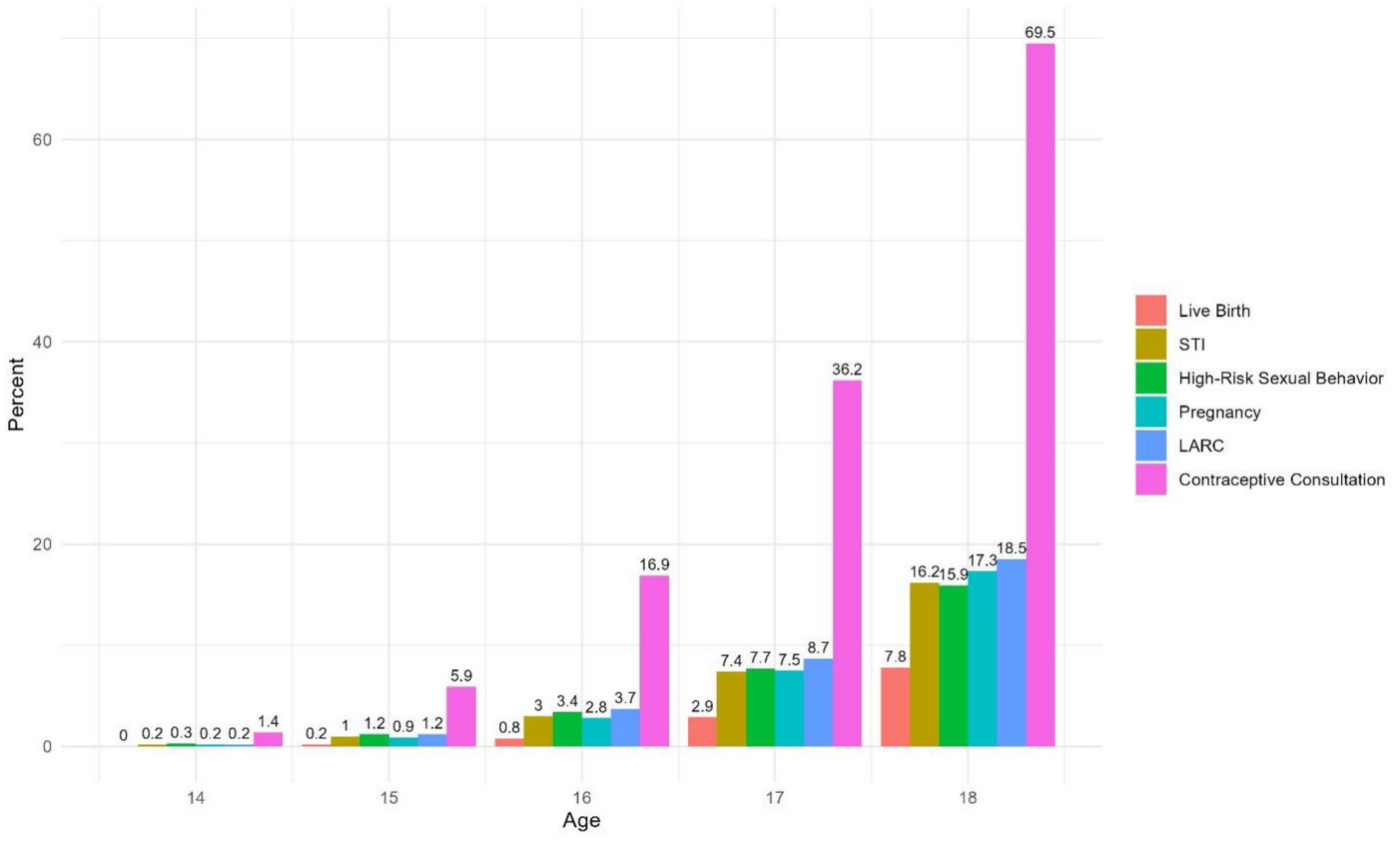
Cumulative incidence of sexual and reproductive health outcomes by age.

Figure 1 depicts age-specific rates of an array of sexual and reproductive health measures. Although pregnancies, STIs, high-risk sexual behavior, births, and LARCs were rare at ages 14 and 15, they increased exponentially with each additional year of age. By age 17, nearly 1 in every 13 adolescent girls involved with CWS had experienced a pregnancy, an STI, and a diagnosis of high-risk sexual behavior. These rates more than doubled by age 18. Upon reaching the age of majority, approximately one in every six adolescent girls (17%) had experienced at least one pregnancy, an STI (16%), and a diagnosis of high-risk sexual behavior (16%). Nearly 8% had experienced a live birth by age 18. In addition, 18% had a healthcare encounter for LARCs, and nearly 70% had a non-LARC contraception consultation by age 18.

### Main regression analysis

Table 2 presents the hazard ratios (HRs) for both the pregnancy and LARC Cox models. Neither current nor previous foster care placement was associated with pregnancy onset. Current foster care placement was associated with a 45% increased hazard of LARC uptake (HR = 1.58, *p* < 0.001) compared with no current or prior placement. However, upon discharge, foster care experience was no longer associated with increased LARC uptake (HR = 1.04, *p* > 0.05). Juvenile legal involvement was associated with a 51% increase in the hazard of pregnancy (HR = 1.51, *p* < 0.001) but was not significantly associated with LARC uptake (HR = 1.09, *p* > 0.05).

**Table 2:** Cox regression models for pregnancy and LARC uptake.

	First pregnancy		First LARC	
	HR	CI	HR	CI
Child race/ethnicity (ref: White)				
Black	1.72	1.57–1.89***	1.07	0.97–1.18
Hispanic	1.72	1.53–1.92***	1.12	1.00–1.27
Other race/ethnicity	1.20	0.92–1.56	0.79	0.59–1.05
Foster care status (ref: no placement to date)				
Currently in foster care	1.02	0.87–1.19	1.58	1.38–1.81***
Previously in foster care	1.10	0.99–1.21	1.04	0.93–1.15
Ever to date juvenile legal referral	1.51	1.37–1.66***	1.09	0.98–1.21
Ever to date mental health diagnoses (not mutually exclusive)				
Post-traumatic stress disorder	0.93	0.84–1.03	1.19	1.08–1.31***
Substance use disorder	1.85	1.67–2.04***	1.48	1.33–1.64***
Attention-deficit/hyperactivity disorder	1.01	0.92–1.11	1.15	1.05–1.26**
Conduct/oppositional defiant disorder	1.10	0.99–1.21	0.93	0.85–1.03
Mood disorder	1.13	1.02–1.24*	1.65	1.49–1.82***
Anxiety disorder	0.90	0.82–0.99*	1.24	1.13–1.36***
Past 6 months CWS contact (not mutually exclusive)				
Related to other reason	1.17	1.02–1.36*	1.11	0.96–1.28
Related to adolescent behavior	1.78	1.59–2.00***	1.27	1.12–1.45***
Related to sexual abuse victimization	1.58	1.26–1.97***	1.70	1.37–2.10***
Prior contraception and pregnancies				
Ever to date non-LARC contraception consultation	1.65	1.51–1.80***		—
Ever to date LARC	0.73	0.62–0.85***		—
Ever to date pregnancy		—	1.79	1.62–1.99***
N subjects	27,944		27,907	
Failure events	2,549		2,502	

Birth year fixed effects included but coefficients not shown.

* *p* < 0.05; **p < 0.01; ***p < 0.001.

Adolescents with a substance use disorder diagnosis had a higher hazard of both pregnancy (HR = 1.85, *p* < 0.001) and LARC (HR = 1.48, *p* < 0.001). In contrast, anxiety disorder diagnosis was negatively associated with pregnancy (HR = 0.90, *p* < 0.05) and positively associated with LARC (HR = 1.24, *p* < 0.001). Mood and ADHD diagnoses were also associated with higher hazard of LARC, but were not associated with pregnancy.

Recent CWS referrals for either sexual abuse or child behavioral issues were associated with increased pregnancy and LARC uptake. Specifically, experiencing alleged sexual abuse in the prior 6 months was associated with a 58% increase in hazard of pregnancy (HR = 1.58, *p* < 0.001) and a 70% increase in the hazard of LARC utilization (HR = 1.70, *p* *<* 0.001). Child behavioral referral was associated with a higher hazard of both pregnancy (HR = 1.78, *p* < 0.001) and LARC utilization (HR = 1.27, *p* < 0.01).

Both LARC uptake and contraceptive consultations were significantly associated with pregnancy, albeit in opposite directions. LARC uptake was associated with a 27% reduced hazard of pregnancy (HR = 0.73, *p* < 0.001) and contraceptive consultation (excluding LARCs) was associated with a 65% increased hazard of pregnancy (HR = 1.65, *p* < 0.001). In addition, a history of pregnancy significantly predicted LARC uptake (HR = 1.79, *p* < 0.001). Lastly, Black and Hispanic females had elevated hazards of pregnancy (HR = 1.72, *p* < 0.001), but there were no statistically significant racial/ethnic differences in LARC uptake.

### Heterogeneity by race/ethnicity

In separate models for White, Black, and Hispanic youths (Table 3), results were broadly similar to the main models, with one key exception. Whereas there was a strong association between recent sexual abuse allegations and pregnancy for White adolescent girls (HR = 1.99, *p* < 0.001), there were smaller, statistically non-significant associations for Black (HR = 1.21, *p* > 0.05) and Hispanic (HR = 1.10, *p* > 0.05) adolescent girls. Sexual abuse was also associated a large increase in the hazard of LARC uptake for both White (HR = 1.89, *p* < 0.001) and Black adolescent girls (HR = 1.76, *p* < 0.05) but not for Hispanic adolescent girls (HR = 1.00, *p* > 0.05).

**Table 3:** Cox regression models for pregnancy and LARC uptake by race/ethnicity.

	White		Black		Hispanic	
	HR	CI	HR	CI	HR	CI
Outcome: first pregnancy						
Foster care status (ref: no placement to date)						
Currently in foster care	0.99	0.78–1.26	1.01	0.79–1.30	1.19	0.81–1.74
Previously in foster care	1	0.86–1.17	1.16	0.99–1.36	1.23	0.94–1.62
Ever to date juvenile legal referral	1.66	1.44–1.91***	1.49	1.27–1.74***	1.23	0.95–1.60
Ever to date mental health diagnoses (not mutually exclusive)						
Post-traumatic stress disorder	0.89	0.78–1.03	1.04	0.87–1.25	0.8	0.62–1.05
Substance use disorder	2.11	1.83–2.42***	1.45	1.21–1.74***	1.86	1.42–2.42***
Attention-deficit/hyperactivity disorder	1.02	0.89–1.16	1.00	0.86–1.18	1.03	0.82–1.30
Conduct/oppositional defiant disorder	1.00	0.87–1.15	1.18	1.00–1.40	1.19	0.92–1.53
Mood disorder	1.25	1.08–1.45**	0.98	0.83–1.15	1.11	0.87–1.43
Anxiety disorder	0.89	0.78–1.02	0.91	0.76–1.09	0.84	0.66–1.07
Past 6 months CWS contact (not mutually exclusive)						
Related to other reason	1.16	0.95–1.42	1.19	0.92–1.53	1.01	0.69–1.49
Related to adolescent behavior	1.77	1.50–2.08***	1.73	1.40–2.14***	1.94	1.45–2.60***
Related to sexual abuse victimization	1.99	1.49–2.67***	1.21	0.77–1.91	1.1	0.60–2.02
Prior contraception						
Ever to date non-LARC contraception consultation	0.84	0.68–1.04	0.74	0.56–0.99*	0.41	0.24–0.69***
Ever to date LARC	1.53	1.35–1.74***	1.75	1.50–2.04***	1.6	1.26–2.02***
N subjects	16,673		6,795		3,566	
Failure events	1,238		857		396	
Outcome: first LARC						
Foster care status (ref: no placement to date)						
Currently in foster care	1.67	1.39–2.02***	1.41	1.09–1.83**	1.65	1.15–2.35**
Previously in foster care	0.97	0.84–1.12	1.17	0.97–1.42	0.92	0.68–1.26
Ever to date juvenile legal referral	1.19	1.04–1.38*	1.03	0.84–1.26	0.81	0.60–1.09
Ever to date mental health diagnoses (not mutually exclusive)						
Post-traumatic stress disorder	1.21	1.07–1.36**	1.15	0.94–1.41	1.22	0.94–1.58
Substance use disorder	1.52	1.34–1.74***	1.25	1.01–1.57*	1.6	1.21–2.12***
Attention-deficit/hyperactivity disorder	1.17	1.04–1.31**	1.08	0.90–1.30	1.23	0.96–1.57
Conduct/oppositional defiant disorder	0.88	0.78–1.00*	0.95	0.77–1.16	1.11	0.85–1.44
Mood disorder	1.61	1.41–1.85***	1.62	1.34–1.97***	1.85	1.40–2.44***
Anxiety disorder	1.31	1.17–1.48***	1.08	0.88–1.31	1.13	0.89–1.45
Past 6 months CWS contact (not mutually exclusive)						
Related to other reason	1.05	0.87–1.26	1.13	0.84–1.51	1.26	0.86–1.85
Related to adolescent behavior	1.21	1.02–1.43*	1.38	1.05–1.80*	1.45	1.02–2.05*
Related to sexual abuse victimization	1.89	1.44–2.47***	1.76	1.13–2.76*	1.00	0.51–1.95
Ever to date pregnancy	1.64	1.43–1.89***	2.00	1.67–2.40***	1.81	1.40–2.34***
N subjects	16,634		6,797		3,565	
Failure events	1,498		619		336	

Birth year fixed effects included but coefficients not shown.

* *p* < 0.05; **p < 0.01; ***p < 0.001.

## Discussion

This study examines predictors of pregnancy and LARC use among adolescent girls with CWS involvement in a large statewide cohort from Pennsylvania. We highlight three findings. First, among those observed until age 18, rates of pregnancy exceeded 20%. Of particular note, this study only considers pregnancies prior to age 18, whereas a large majority of teen pregnancies overall occur at ages 18 and 19. Moreover, because we are unlikely to observe pregnancies that result in miscarriage or abortion in the first trimester, these estimates are highly conservative. Thus, our estimate that one in six adolescent girls involved with CWS will experience pregnancy before reaching the age of majority is highly concerning. This finding signals that, despite broad declines in adolescent pregnancy within the general US population, the phenomenon remains comparatively common among adolescent girls involved with the child welfare system. Others have similarly observed that, as overall rates of teen pregnancy have declined, the social and economic disadvantages of pregnant adolescents have become more pronounced. Indeed, we show that, even within the population of adolescent girls involved with CWS, pregnancies are concentrated among girls with substantial behavioral health concerns, including CWS involvement due to behavioral health problems, involvement with the juvenile legal system, and diagnosed substance use disorder.

Second, we identify that recent sexual abuse allegations were strongly associated with pregnancy, especially for White adolescents. Many prior studies have linked historical sexual abuse to a higher incidence of pregnancy, focusing on how sexual abuse affects behaviors related to substance use, choice of romantic or sexual partners, and decision-making. However, the temporal proximity of sexual abuse allegations to pregnancy in our study raises questions about the share of pregnancies that may be a direct result of abuse. This study cannot determine the other party involved in the pregnancies, but future research should consider the possibility and implications of pregnancies resulting from sexual abuse.

Third, despite a bivariate association between foster care and early pregnancy, there was no association between these measures in multivariate models that accounted for behavioral health factors. Given that behavioral health is a risk factor for both foster care placement and early pregnancy, it is perhaps expected that controlling for untreated or undertreated behavioral health problems would attenuate the association between foster care and early pregnancy. However, it also highlights the importance of improving the efficacy of CWS in addressing behavior concerns—particularly externalizing behavior like substance use and delinquency—both in and out of foster care. We also note that current foster care placement was associated with higher rates of LARC, which is highly effective in preventing pregnancy. It is not clear why foster care would increase LARCs. However, it is possible that this association reflects improved detection and treatment of care needs, given that youths in foster care are required to have routine medical exams and thus receive more frequent preventive and treatment-oriented healthcare services.

## Limitations

Study data reflect a single state, Pennsylvania, which may limit generalizability of the findings. Pennsylvania has some unique features. First, its general protective services system serves youths with behavioral health concerns, potentially resulting in a different composition of adolescent girls in the CWS than other states. Second, there are challenges to identifying the timing of pregnancy onset due to variability in observation of a live birth and incomplete use of the trimester-specific ICD-10 subcodes. Thus, there is some uncertainty surrounding age at first pregnancy. Medicaid claims data are more limited than Electronic Health Records, which were not available for this study. Third, our sample was restricted to adolescents on Medicaid; although this captures nearly all adolescents in foster care and most who exited to adoption or guardianship, it excludes some youths who remained in or return home following CWS involvement. Lastly, because elective abortions cannot be billed to Medicaid and healthcare services may not be sought for early-stage miscarriages, we are unlikely to accurately count pregnancies that result in miscarriage or abortion within the first trimester. Hence, our estimated rate of pregnancy within this population is highly conservative.

## Implications

Adolescent girls involved with the child welfare system remain highly vulnerable to early pregnancy, which can disrupt education and other critical milestones and elevate risk for intergenerational involvement in the child welfare system. Risks are especially elevated among those with behavioral health problems, including involvement in the juvenile legal system and substance use disorders. Increasing access to evidence-based behavioral health services, which are eligible for federal match funding under the Family First Prevention Services Act, may mitigate some risk factors for early pregnancy and parenthood in this population. However, adolescent girls may also benefit from improved screening for sexual risk behaviors and referral to contraceptive services, including access to LARCs. Alternatively, it is possible that referrals and screening practices are sufficient, but follow-up is limited by youth preferences or structural barriers. Additional research is needed to understand youth preferences, concerns, and barriers to accessing care, as well as provider views and practices. Regardless, LARCs are an important option for this population, given that they may face both interpersonal and structural barriers to reliably using other pregnancy prevention methods.

## Data Availability

The data analyzed in this study is subject to the following licenses/restrictions: Data were provided under a business associates agreement with Pennsylvania DHS and cannot be shared. Requests to access these datasets should be directed to the corresponding author.

## Appendix A. Codes used for main measures.

**Table:**

	ICD 10 diagnosis code	ICD 9 diagnosis code	Current procedural terminology (CPT)/healthcare common procedure coding system (HCPCS) codes
Pregnancy	All O codes; Z33; Z34; Z36; Z37; Z39; Z3A; A34; F53; Z64.0; Z64.1	637–679; V22–V24; V27; V28; V91; V61.5; V61.6; V61.7	—
Long-acting reversible contraception	Z30.014; Z30.017; Z30.430; Z30.431; Z30.433; Z30.46; Z97.5	V25.02; V25.11; V25.13; V25.42; V25.43; V25.5; V45.51	58300; 58301; 11981; 11982; 11983; J7296; J7297; J7298; J7300; J7301; J7307
Other contraceptive consultation	Z30.011; Z30.012; Z30.013; Z30.015; Z30.016; Z30.018; Z30.019; Z30.02; Z30.09; Z30.2; Z30.40; Z30.41; Z30.42; Z30.432; Z30.44; Z30.45; Z30.49; Z30.8; Z30.9	V25.01; V25.03; V25.04; V25.09; V25.12; V25.2; V25.3; V25.4; V25.40; V25.41; V25.49; V25.8; V25.9	—
High-risk sexual behavior	A54; A55; A56; A58; A59; Z20.2; O98.1; O98.2; O98.3; O98.4; Z72.5; Z11.3; Z11.4; Z11.51; Z71.7	098; 099.1; 099.2; 099.41; 099.5; 131; V01.6; 647.0; 647.1; 647.2; V69.2; V73.81; V73.88; V73.98; V74.5; V65.44; V65.45	—
Sexually transmitted infections	A54; A55; A56; A58; A59; Z20.2; O98.1; O98.2; O98.3; Z11.3; Z11.4; Z11.51; Z71.7	098; 099.1; 099.2; 099.41; 099.5; 131; V01.6; 647.0; 647.1; 647.2; V73.81; V73.88; V73.98; V74.5; V65.44; V65.45	—
